# Serious absenteeism amongst Pakistani school and university girls during menstruation: Is this a neglected threat to already deteriorating girls' education in the country?

**DOI:** 10.1016/j.lansea.2022.100072

**Published:** 2022-10-07

**Authors:** Syeda Tayyaba Rehan, Hassan ul Hussain, Mohammad Mehedi Hasan

**Affiliations:** a Dow University of Health Sciences, Karachi, Pakistan; b Department of Biochemistry and Molecular Biology, Faculty of Life Science, Mawlana Bhashani Science and Technology University, Tangail, Bangladesh

According to the 2018 data, 22.5 million children in Pakistan are not enrolled in school, making it the worst country in the world for its educational achievement.^1^ Due to the larger gender disparity, irrational social and moral conventions, and high rates of early marriages in developing nations like Pakistan, females suffer the most when it comes to the barrier to education or deprivation of any other fundamental requirements of living. Surprisingly, just 13% of the nation's girls are enrolled in school by grade nine.^1^ The majority of girls are compelled to leave school at menarche because in many rural and in a small fraction of urban areas, the onset of menstruation is seen as an indication of marriage. From grade six to nine nearly 59 percent of girls dropout of school in Pakistan.^1^ There exist numerous roots for this long-going deterioration of women's education in the country, here we aim to explore the absenteeism of school and university students during their menstrual cycles, which ultimately subsides their academic interests and creates an educational gap when compared with the rest of their peers.

One of the most underreported problems affecting girls' education in poor nations is the absence of female students from schools and institutions during their menstrual cycles.^2^ According to estimates, underprivileged girls miss up to two consecutive school days each month due to menstruation, which represents a significant loss of their educational time and hinders their overall academic performance quality.^3^ Numerous Asian nations, including India, Indonesia, Bangladesh, Japan, and Nepal, performed surveys that revealed that 40%, 11%, 41%, 11.9%, and 32.7% of schoolgirls skip courses during their periods, respectively.^4^, ^5^, ^6^, ^7^, ^8^

A cross-sectional survey from five Pakistani girls' schools revealed that 55% of the girls missed class while they were on their periods.^9^ United Nations International Children's Emergency Fund (UNICEF) broke their silence on menstrual hygiene in April 2017 and declared that due to poor menstrual hygiene and lack of affordable sanitary facilities, most Pakistani girls miss out on school and work. Due to the prevalent prejudices and misunderstandings about menstruation in society, these girls are forcibly barred from attending school at the beginning of their menstrual events and even drop out of school at menarche^2^

Menstrual hygiene management (MHM) organisations claimed in 2017 that girls skip school during their periods as a result of societal pressures and the imposition of false notions, which has a significant negative impact on their scholastic performance.^2^^,^^10^ University students reported no variation in skipping their courses on the days of their periods, which is similar to the absenteeism of schoolgirls. A survey conducted at the Rawalpindi Medical University disclosed that 19.3% of the girls miss at least two days of their menstruation every month. The study further reported that 14.8% of the girls did not attend classes during their menstruation due to the fear of staining their clothes and end up being ashamed about it, while a large proportion of girls (54.5%) were found to miss their lessons due to the pain and cramps associated with the cycle.^3^

40.6% of the university girls said that their work routine was affected and 19.3% reported that they could not perform well at educational platforms during their cycle days which led them to miss the classes more often.^11^ Students also reported suffering from premenstrual syndrome (PMS) symptoms, which were particularly more common amongst hostellers as compared to the day scholars due to the stress of living away from home.^12^ Further, a survey of 145 medical students recorded that 20.7% of the students remained absent from classes during their menstruation phase due to PMS symptoms.^13^

Menstruation-related hurdles had caused a serious level of educational absenteeism amongst adolescent girls. Mainly the factors like poor sanitation facilities at institutes, physical discomfort, social pressure, and fear of getting ashamed contributed to the girls' absence from institutes, making them more prone to already prevailing ill social values.^2^^,^^9^^,^^10^ Girls' monthly absence at this rate is a worrying trend that has gone unchecked for far too long. Although a few of the country's organisations began investigating the issue, no formal action plan has yet been established by institutions to address this problem. We suggest that there is a dire need to improve the sanitary facilities in institutes. A few policies that should be mandated at all institutions include the availability of hot pads and painkillers, as well as hygienic ladies' restrooms. It is important to start awareness campaigns on menstrual taboos to inform young women about the risks of upholding false notions regarding this natural bodily process. The societal stigmas associated with menstruation should also be busted, and mothers should be counselled to encourage rather than forbid their daughters from attending school when they are on their periods. Moreover, the Government should channel efforts to introduce initiatives where male and female instructors actively encourage females of all ages to openly discuss their menstruation challenges rather than being stigmatised for discussing their physical changes.

## Contributors

Syeda Tayyaba Rehan: Conceptualization, Writing – original draft. Hassan ul Hussain: Writing – original draft. Mohammad Mehedi Hasan: Conceptualization, Writing - review & editing.

## Declaration of interests

None.
